# Effect of sex and milk replacer with or without supplemental carnitine and arginine on growth characteristics, carcass, and meat quality of artificially reared low-birth weight pigs

**DOI:** 10.1093/jas/skae122

**Published:** 2024-06-01

**Authors:** Johannes G Madsen, Michael Kreuzer, Paolo Silacci, Giuseppe Bee

**Affiliations:** Department of Veterinary and Animal Sciences, University of Copenhagen, Denmark; Department of Environmental Systems Science, ETH Zürich, Switzerland, Switzerland; Swine Research Group, Agroscope, Switzerland; Swine Research Group, Agroscope, Switzerland

**Keywords:** low-birth weight, meat quality, muscle fiber, muscle metabolism, piglets, supplements

## Abstract

This study compared milk replacer either remaining unsupplemented (CON) or supplemented with 0.5 g L-carnitine plus 16.7 g L-arginine/kg (CarArg) and fed to 48 low-birth weight (**L-BtW**) artificially reared piglets (24 per group) from days 7 to 28 of age. Eight farrowing series were needed to complete the study. On day 28, the lightest piglets were slaughtered, and the heaviest pigs were weaned. The heaviest pigs were weaned on day 28 and offered free access to a starter (weaning to 25 kg body weight [**BW**]), grower (25 to 60 kg BW), and finisher diet (60 to 96 kg BW on day 170 of age). After euthanization on days 28 and 170, blood was sampled for assessment of serum metabolite and hormone concentrations, and the semitendinosus muscle (**STM**) was weighed, and later subjected to enzyme activity analysis and assessment of myofiber characteristics. In the 170-d-old pigs carcass and meat quality traits were assessed. Growth data were analyzed **accordingtoatwo**-**way** analysis of variance (ANOVA), with dietary treatment and farrowing series as fixed effects, while remaining data were analyzed with dietary treatment, sex, their interaction, and farrowing series as main factors. Dietary treatments affected (*P* ≤ 0.049) muscle enzyme activity at both day 28, with greater citrate synthase (**CS**) and LDH activities and lower HAD:CS ratio in STM light portion, and lower LDH:CS ratio in STM dark portion, and 170 of age with lower HAD:CS ratio. In the starter period, CarArg pigs had greater average daily gain (*P* = 0.021) and average daily feed intake (*P* = 0.010). At slaughter, these pigs had lower (*P* = 0.013) glucose and greater (*P* = 0.022) urea serum concentrations. However, supplementing the milk replacer with carnitine and arginine had no long-term effects on growth performance, carcass composition, and meat quality of L-BtW pigs. In addition, muscle morphology and myofiber-related properties remained unaffected by the supplementation.

## Teaser

Due to selection for large litters, modern sows now give birth an increased number of runts that potentially require extra care. Concomitantly the number of low-birth weight (L-BtW) piglets increases. Managing L-BtW piglets, which display poor lifetime performance, carcass composition, and meat quality, has become a major challenge for pig producers. Therefore, alternative rearing strategies are necessary to accommodate the special needs and requirements of this particular subset of piglets. One such strategy is artificial rearing in rescue decks, protecting the most vulnerable piglets of a litter. Feeding exclusively with milk replacers provides an opportunity for the supplementation of specific nutrients present in low concentrations in sow’s milk. The experimental objective was to show whether supplementation with extra carnitine and arginine during artificial rearing had long-term effects in the subsequent fattening period.

## Introduction

Pig producers are currently facing the challenge of managing an increased number of low-birth weight (**L-BtW**) growth-impaired piglets from hyperprolific sows (Campos et al., 2012; Ward et al., 2020). This issue is the result of decades of selection for sows with more live-born piglets (Martineau and Badouard, 2009). As a result, the variation within litters concerning birth weight has increased (Milligan et al., 2002; Riddersholm et al., 2021; Knap et al., 2023). Additionally, the crowded conditions within the uterus have led to intrauterine growth restriction (**IUGR**), which has, in turn, increased the number of L-BtW piglets, further supported by research findings from (Foxcroft et al., 2007; Riddersholm et al., 2021). The L-BtW piglets are more prone to early postnatal death (Tuchscherer et al., 2000; Milligan et al., 2002; Peltoniemi et al., 2021), have impaired growth (Rehfeldt and Kuhn, 2006), show reduced function and capacity of the small intestine (Zheng et al., 2018), display higher level of metabolic and oxidative stress in the pre-weaning period (Novais et al., 2020), as well as displaying disturbances in the glucose and lipid metabolism (Goodarzi et al., 2021) compared with their normal birth weight littermates. Furthermore, a poorer carcass composition, as exemplified by lower lean meat content and greater mean backfat depth, compared with that of their larger littermates has previously been observed (Gondret et al., 2006). The latter is likely the consequence of smaller muscles, particularly mediated by a reduced number of myofibers of L-BtW compared with heavier BtW piglets, and outcome of IUGR (Lefaucheur et al., 2003; Bérard et al., 2010b). The proposed link between these parameters suggests that the observed increase in fat deposition, due to a diminished potential for lean growth, can be attributed to the capacity of individual myofibers for protein accretion and hypertrophy (Bee, 2007). Recent studies have shown that early artificial rearing combined with the provision of milk replacers supplemented with either L-carnitine or L-arginine were beneficial for maturation and activation of the protein synthesis pathway, respectively, in the semitendinosus (**STM**) muscle (Madsen et al., 2018a, 2018b). However, there has been no clear indication that one supplement has an advantage over the other, probably because the two supplements act in different pathways, respectively, L-carnitine acts via beta-oxidation of fatty acids (Stephens et al., 2007; Keller et al., 2011, 2012) and L-arginine is involved in enhancing protein synthesis (Kim and Wu, 2004; Yao et al., 2008; Wu et al., 2009). With respect to the immediate attention needed concerning growth rate, the focus has been on the period from birth to weaning. Hence, it is unknown whether the impact of early artificial rearing in rescue decks and diet supplementation would persist into the starter (weaning to 25 kg body weight [**BW**]), grower (25 to 60 kg BW), and finisher (60 kg BW to slaughter) periods. In addition, it is also unknown whether artificial rearing and milk replacer supplementation would affect carcass characteristics and meat quality traits of pigs slaughtered at a common age and weight.

The first objective in the present study was, therefore, to investigate if a combination of L-carnitine and L-arginine (CarArg) supplementation compared with an unsupplemented control (CON) would elicit a beneficial effect on muscle development of L-BtW piglets and increase growth rate during the nursing period. Considering the lack of carryover data into the postweaning growth period, the second objective was to investigate if early artificial rearing of L-BtW fed a milk replacer supplemented with the two compounds would improve growth, carcass characteristics, and meat quality even after termination of the supplementation at weaning. The hypothesis was that a diet combining L-carnitine and L-arginine for artificially reared L-BtW piglets would either synergistically or additively promote both the maturation and protein synthesis in the STM muscle, leading to improved growth performance.

## Materials and Methods

### Animal ethics

All procedures involving animals used in this study were approved by the Swiss Cantonal Committee for Animal Care and Use (2014_64_FR). Every day prior to feeding, the health condition of all piglets was observed. In the case of diarrhea, the piglets were treated with either Borgal (Virbac group, Carros, France) or Colivet (Prodivet Pharmaceuticals, Hagbenden, Belgium).

### Animals, treatments, and experimental conditions

The experiment was conducted at Agroscope Swine Research Facility, Posieux, Switzerland. The experiment was divided into two phases: phase one investigated the growth performance and muscle and intestinal development of artificially reared L-BtW piglets from 7 to 28 d of age fed either an unsupplemented milk replacer (CON) or a milk replacer supplemented with a combination of L-carnitine and L-arginine (CarArg). In the second phase, selected piglets of phase 1 were used to investigate the carryover effects of this rearing strategy on postweaning growth characteristics, carcass composition, and meat quality. In the first phase, a total of 48 piglets (24 females and 24 castrated males) from Swiss Large White sows (parity 1 to 7) were used. Sows were artificially inseminated with pure Swiss Large White boars. Within farrowing series, the semen of the same boar was used for insemination. To achieve the needed animal count, eight farrowing series were conducted. Series 1, 3, 5, and 8 each contributed 4 pigs, while series 2, 4, 6, and 7 each contributed 8 pigs. Males were castrated within the first 4 d after birth. Piglets originated from large litter sows (average litter size of total born piglets: 17.1 ± 1.56 and average litter BtW: 1.23 ± 0.327 kg [mean ± standard deviation]). The birth weight (**BtW**) of the piglets used in the study averaged 1.04 ± 0.110 kg, which was at least 300 g lower than the average BtW of piglets born from the Agroscope sow herd in the previous year. On the day of birth, the piglets were weighed, and their crown-to-rump length (**CRL**) was measured to calculate body mass index (**BMI**: BtW × CRL^2^) and **ponderal index** (PI: BtW × CRL^3^). Piglets with a similar BtW and day 7 BW were weaned in pairs on day 7, blocked by BtW, and randomly allocated within blocks to either CON (BtW: 1.04 ± 0.119 kg and BW on day 7: 1.78 ± 0.246 kg) or CarArg (BtW: 1.05 ± 0.102 kg and BW on day 7: 1.75 ± 0.269 kg). Sex was balanced between the blocks, with 12 females and 12 castrates in each dietary treatment group. Piglets were reared in customized rescue decks (0.49 m × 1.1 m, semi-slatted floor) for 21 d. From each piglet pair, the one displaying the lower BW on day 21 of age was sacrificed on day 28 of age. The same day, the remaining 12 (5 females and 7 castrates) CON- and 12 (7 females and 5 castrates) CarArg piglets (average BtW: 1.08 ± 0.090 kg) were moved to group pens and reared together with other, conventionally reared, L-BtW piglets (n = 24, average BtW: 1.08 ± 0.110 kg) born from hyperprolific sows (average litter size: 16.5 ± 1.56 kg, average litter BtW: 1.30 ± 0.158 kg). Artificial rearing of the piglets as well as the postweaning trial were conducted within the same piggery facility.

### Diet and feeding regime

In the first phase of the experiment, piglets were offered a commercial milk replacer (Provimi Neopigg Rescuemilk 2.0, Provimi BV, Rotterdam, the Netherlands), whose main ingredients were whey powder, whole milk powder, and complete milk protein. Their analyzed gross chemical nutrient and energy composition is presented in Supplementary Table S1. The milk replacer for the CarArg group was supplemented with L-carnitine (Carniking, Lohmann Animal Health, Cuxhaven, Germany) and L-arginine HCI (Evonik Degussa GmbH, Hanau, Germany), with final concentrations of 0.5 g/kg diet and 16.7 g/kg diet, respectively. This dosage was chosen based on Arg concentration in sow milk from the research herd (Madsen et al., 2018b) as levels above this concentration have altered gene expression in the muscle of pigs (Keller et al. 2011), and previously defined effective dosages (Kim and Wu 2004). To make the CON and CarArg diets isonitrogenous, a corresponding amount of L-alanine (Evonik Degussa GmbH, Hanau, Germany) was added to the CON milk replacer. The milk replacer was prepared fresh every morning and contained 130 g DM/kg. In addition, the CarArg and CON creep feed diets were premixed with the same experimental levels of either L-carnitine and L-arginine, or L-alanine. Creep feed was offered to the piglets from days 21 to 28 of age. The piglets had ad libitum access to the respective milk replacers, creep feed, and water. The piglets were weighed on the day of birth and on days 2, 7, 14, 21, and 28 of age.

In the second phase of the experiment, the pigs were fed a standard pelleted starter (weaning to 25 kg BW), grower (25 to 60 kg BW) and finisher diet (60 kg BW to slaughter) until slaughter at day 170 of age. The Supplementary Table S1 displays the ingredients and analyzed composition of the three diets. In each farrowing series, 4 to 8 pigs were initially kept in a pen for weaned piglets until they reached 20 kg BW. Following this, they were moved to a grower-finisher pen, where they stayed until slaughter. To maintain a consistent space allowance of 1.2 m² per pig across all series, unrelated grower-finisher pigs were added to the pens as necessary. This ensured uniformity in living conditions throughout the study. Each pen was equipped with an automatic feeder and a pig recognition system (Schauer Maschinenfabrik GmbH. & Co Kg, Prambachkirchen, Austria) monitoring the individual daily feed intake and feeding behavior as previously described by Bee et al. (2008). The first 3 wk after weaning, piglets were too small to be able to eat from the automatic feeder, and therefore the starter diet was offered in troughs. During this period feed intake and time spent eating were not recorded. Subsequently, the individual feed intake of each visit as well as time spent eating were recorded.

### Measurements and sampling at slaughter

On day 28, half of the piglets were anesthetized with an isoflurane-oxygen mixture [4% vol/vol] and euthanized by exsanguination in the same facility as they were raised. Feed was withdrawn 10 h prior to slaughter. Blood samples were collected at exsanguination into 9 mL vacutainers (Vacuette, Greiner Bio-one GmbH, Austria) one with and one without heparin as an anticoagulant for plasma and serum, respectively, and centrifuged at 1,000 × *g* for 15 min at 20 °C. Serum was transferred to 2 mL microtubes (Treff AG, Degersheim, Switzerland) and stored at −20 °C until further analysis. After exsanguination, the carcasses were opened to collect and weigh the heart, liver, kidneys, spleen, lungs, and adrenal glands. The hot carcass was then weighed and split along the midline including the head. From the head the total brain was collected and weighed. Within 15 min after exsanguination, the whole semitendinosus muscle (STM) from the right carcass side was excised and weighed, and the length and circumference at the midpoint of the muscle were measured. The circumference was used to calculate the area of the STM. The STM was then divided into its dark (**STMd**) and light (**STMl**) portions. From each portion, a subsample cut longitudinally to the muscle fiber was removed and snap frozen in liquid nitrogen-cooled 2-methylbutane, which was cooled in liquid nitrogen and then stored at −80 °C until histochemical and enzyme activity analyses were performed. In addition, the intestine was emptied, and the length and weight of the small and large intestines were measured. From the small intestines, one piece from the duodenum, jejunum, and ileum was collected and immersed in 4% formalin for later histological assessment of villi height and width and crypt depth. Next, the left carcass sides were cooled at 3 °C for 1 d. The cooled carcasses were then cut into smaller pieces and homogenized in a primary step with a meat mincer (R 2 version “A”, Robot-Coupe SNC, Montceau en Bourgogne Cedex, France) and frozen for at least 1 wk at −80 °C. The minced frozen carcass samples were then freeze-dried (Delta 1-24 LSC, Christ, Osterode am Harz, Germany), then frozen with liquid nitrogen and homogenized with a Grindomix GM 200 (Retsch, Haan, Germany) for later chemical analysis.

On day 170 of age, and after a fasting period of 15 h, the remaining half of the pigs were slaughtered in the research abattoir of Agroscope, Posieux. The animals were moved in pairs from the pen to the stunning area (100 m), rested for at least 10 min, and were stunned with a CO_2_:O_2_ (87:13) gas mixture for 150 s using a CO_2_ stunner (MPS meat processing systems, Lichtenvoorde, The Netherlands). At exsanguination, blood samples were collected in two 9 mL vacutainers (Vacuette), one with and one without heparin as an anticoagulant for plasma and serum, respectively. The samples were then centrifuged and stored like those obtained from the 28-d-old piglets. Subsequently, the carcasses were scalded at 62 °C, de-haired, eviscerated, and split along the midline including the head. The weights of the hot carcass, and with the same procedure as described with the 28-d-old piglets organs were removed and weighed. In addition, the whole right STM was excised and weighed, as well as the respective weights of the emptied small and large intestines, and were measured. Furthermore, the length and circumference of the STM and the length of the small and large intestines were measured. After being chilled for at least 24 h at 2 °C, the cold carcasses were weighed again. Subsequently, the left carcass side was dissected into the primary cuts (loin, ham, shoulder, and belly) and back fat thickness was determined at the 10th rib level of the Longissimus thoracis (**LT**). Lean meat percentage was derived by adding the weights of the denuded loin, ham, and shoulder, and then expressing this sum as a percentage of the weight of half the cold carcass. Similarly, the percentage of carcass fat was determined by summing the weights of fat from the loin, shoulder, and ham, and expressing this total as a percentage of the half-cold carcass weight. Similarly, the belly percentage was calculated by taking the weight of the belly and expressing it as a percentage of the weight of half the cold carcass. Samples of the LT were collected between the 10th and 13th ribs, freed from subcutaneous adipose tissue, and divided into five 1.5 cm thick slices.

### Measurement of meat quality traits

The change in LT temperature and pH was monitored 45 min, 3 h, and 24 h postmortem at the 10^th^ rib level using a pH meter (WTW pH 197-S, WTW, Weilheim, Germany) equipped with an insertion glass spearhead electrode (Metrohm, 6.0226.100) combined with a temperature probe (Pt 1000, Metrohm AG, Herisau, Switzerland). Chops were used to measure intramuscular fat content, objective color, drip loss, thaw loss, and cooking loss. Meat color was assessed using a Chroma Meter CR-300 (Minolta, Dietikon, Switzerland) with a D65 light source and 0° viewing angle geometry according to the reflectance coordinates (CIE L*, a*, b*) after exposing the muscle surface to ambient air for 20 min. Two of the five slices were weighed, vacuum packaged, kept at 4 °C for 72 h, and then stored at −20 °C. Later the frozen samples were thawed for 24 h at 2 °C and then weighed to determine thaw loss. After 1 h at room temperature, the chops were cooked on a preheated (190 to 195 °C) grill plate (Beer Grill AG, Zurich, Switzerland) to an internal temperature of 69 °C recorded by a thermometer probe. The chops were cooked for 1.5 min on one side, then turned and cooked for 3.5 min, and finally 2 min on the initial side. Slices were reweighed to determine cooking loss. Shear force was determined as described by Pardo (Pardo et al., 2013). In brief, when meat samples reached room temperature (approx. 2 h after cooking), shear force was determined (five cores per sample) using a Stable Micro System TA.XT2 Texture Analyzer (Godalming, Surrey, UK) equipped with a 2.5-mm-thick Warner–Bratzler shear blade. The shear force values were expressed as the average of the five measurements. Other two of the five slices were used to assess 24 h drip loss using the plastic bag method described by Honikel (Honikel, 1998).

### Chemical analysis of diets, carcasses, and meat

The dry matter (**DM**) content of samples from the milk replacer, pelleted diets, and carcasses of the 28-d-old piglets was determined by drying samples at 105 °C for 160 min. Subsequently, samples were analyzed for total ash content with a thermogravimetric analyzer (Leco TGA-601, Leco Corporation, St. Joseph, MI, USA). Gross energy content was determined by adiabatic bomb calorimetry (AC600 Semi-Automatic Calorimeter, Leco Corporation, St. Joseph, MI, USA). The nitrogen content of the milk replacer and pelleted diets was analyzed according to the Dumas method using the automated CNS elemental analyzer (TruMac Series, Leco Corporation, St. Joseph, MI, USA), whereas for the carcasses, the Kjeldahl method was used (Kjeltec 2400/2460; AOAC, 2012). The CP content was calculated as 6.25 × nitrogen. To determine the crude fat content of the milk replacer, pelleted diets, carcasses, and LT samples (IMF) samples were analyzed by acid hydrolysis (10% HCl solution; Hydrotherm HT6, C. Gerhard, Königswinter, Germany) followed by a petroleum ether extraction (Speed Extractor 916, Büchi Labortechnik AG, Flawil, Switzerland). The amino acid composition of the milk replacer and starter, grower, and finisher diets was analyzed by HPLC using the 2695 Alliance Separation Module coupled to the Alliance Column Heater and the 2475 Multi-Lambda Fluorescence Detector (Waters Corporation, MA, USA) according to manual description (Waters AccQ Tag Chemistry Package 052874 TP, rev. 1). All analyses were performed as biological duplicates.

### Analysis of blood plasma and serum

Blood serum urea (Art. no. 147116 Greiner Diagnostic, Bahlingen, Germany), non-esterified fatty acids (**NEFA**, Art. no. FA 115, Randox, Crumlin, UK), and glucose (Art. no. 244L, Biotechnica instruments, Rome, Italy) were analyzed according to the manufacturers’ protocols on a BT1500 analyzer (Biotechnica instruments, Rome, Italy). Enzyme-linked immunosorbent assay (ELISA) kits (Cloud-Clone Corp., Houston, TX, USA) were used to determine the blood plasma concentrations of growth hormone (**GH**, CEA044Po), insulin-like growth factor 1 (IGF-1, CEA448Po), and insulin (SEA050Po) in 170-d-old pigs by following the manufacturers’ protocols. With respect to IGF-1, five samples of the CON group and two samples of the CarArg group displayed concentrations below the detection levels and were not included in the statistical analysis.

### Histochemical and enzyme activity analysis of the semitendinosus muscle

The myofiber characteristics of the STM were assessed in the 28-d-old piglets by applying histochemical procedures as described previously by Bérard and Bee (2010). In brief, 10 μm thick cross-sections of the STMd and STMl were cut perpendicular to the fiber direction at −20 °C in a cryotome (Shandon cryotome, Shandon Inc., Pittsburg, PA, USA). Two sections per sample were stained for the determination of myofibrillar ATPase activity after acid pre-incubation at pH 4.37. The stained sections were observed at 20 X magnification using a BX53 microscope in transmitted light mode (Olympus Optical Co., Hamburg, Germany) equipped with a high-resolution digital camera (ColorView12, Soft Imaging System GmbH, Münster, Germany) and captured as TIFF images. 1n both sections of the STM, type I and type II myofibers were identified based on their dark and light staining, respectively. The areas and numbers of the type I and type II myofibers in the STMd and STMl were determined with the analySIS software 3.0 image (Soft Imaging System GmbH, Münster, Germany). The total number of myofibers (TNF) was estimated by counting the total number of type I and type II fibers in a defined area (0.14 mm^2^) and extrapolating these numbers to the total muscle area. One STMd sample from the CON and one STMd sample from the CarArg group could not be adequately stained and were therefore excluded from the statistical analysis of myofiber-related properties.

To characterize the overall oxidative capacity (tricarboxylic cycle), lipid β-oxidation and glycolytic potential of the STMd and STMl, the activities of citrate synthase (**CS**; EC 4.1.3.7), β-hydroxyacyl-CoA dehydrogenase (**HAD**; 1.1.1.35), and lactate dehydrogenase (**LDH**; EC 1.1.1.27) were measured as described in Madsen et al., 2018b. Prior to activity assessment, 50 to 100 μg of STMd and STMl stored at  80 °C were lysed in 500 μL of CelLytic MT buffer (Sigma-Aldrich Chemie GmbH, Buchs SG, Switzerland) supplemented with Complete TM Inhibitor Cocktail Tablets (F. Hoffmann-La Roche Ltd., Rotkreuz, Switzerland) using the Precellys Lysing Kit CK 14 (Bertin Technologies, Montigny le Bretonneux, France) and Minilys Tissue Homogenizer (Bertin Technologies, Montigny le Bretonneux, France) at maximum speed for 40 s. The lysed samples were then centrifuged at 12,000 × *g* for 10 min at 4 °C, and the supernatant was assessed for the protein concentration using the Pierce Coomassie Plus (Bradford) Assay Kit (Thermo Scientific, Wilmington, DE, USA). The activities of LDH and CS were determined with the LDH and CS BioVision Activity Colorimetric Assay Kits (Biovision Incorporated, CA, USA) following the manufacturer’s protocol. The activities of LDH and CS were monitored at 450 nm for 10 min and at 412 nm for 30 min, respectively, with a microplate reader (Biochrom Asys UVM340, Biochrom Ltd., Cambridge, UK), and the readings were analyzed with MicroWin 2000 software (Hidex, Turku, Finland). The HAD activity was determined in a solution containing 97 mM/L potassium phosphate, 0.09 mM/L S-acetoacetyl-coenzyme A, 0.1 mM/L β-nicotinamide adenine dinucleotide after incubation for 3 min at 37 °C. The activity was measured at 340 nm with a spectrophotometer (Biochrom WPA Biowave II, Biochrom Ltd., Cambridge, UK) at initiation (0 min) and 3 min after the incubation. The enzyme activities were expressed as units/mg of protein, where one unit is defined as the amount of enzyme hydrolyzing 1 mM substrate/min.

### Histochemical analysis of the small intestine

For the determination of the morphology of the duodenum, ileum, and jejunum of the 28-d-old piglets transverse sections of each part of the small intestine were adjusted to fit the processing cassettes of the Tissue Processing Center TPC 15 (MEDITE GmbH, Burgdorf, Germany). The 3-μm thick transverse paraffin sections were stained with hematoxylin-eosin using the Leica ST5020 slide stainer (Leica Biosystems, Muttenz, Switzerland). Average height and width of the intestinal villi and crypt depth were determined in at least 60 longitudinal cut villi and adjacent crypts per sample.

### Determination of the phosphorylation of the mammalian target of rapamycin-signaling pathway in the semitendinosus muscle

To investigate the activity of the mammalian target of rapamycin (mTOR) in the STM of 28-d-old piglets, the phosphorylation of Ser_2481_ residue was analyzed as described in Madsen et al., 2018b. First, for the immunoblotting, 100 μg of tissue from each of the frozen STMd and STMl samples were transferred to individual CK 14 tubes (Bertin Technologies, Montigny le Bretonneux, France) containing 400 μL of Cell Lytic MT Cell Lysis Reagent (Sigma-Aldrich Chemie GmbH, Buchs SG, Switzerland) supplemented with a protease-phosphatase inhibitor cocktail (Cell Signal Technology, Danvers, MA, USA). Homogenization was then performed by bead beating using the Minilys device (Bertin Technologies, Montigny le Bretonneux, France) with maximal agitation for 1 min. The homogenate was then further centrifuged at 10,000 × *g* for 10 min at 4 °C. Subsequently, 100 μg of protein in the supernatant was subjected to electrophoresis on a 7% polyacrylamide gel and then transferred by electrophoresis to a polyvinylidene difluoride membrane (Western Bright PVDF L-08004-010, Witec, Luzern, Switzerland). To detect the amount of phosphorylated mTOR, the gels were incubated for 12 h at 4 °C with a rabbit polyclonal anti-phospho mTOR primary antibody (Ser_2481_) (Cell Signal Technology, Danvers, MA, USA) diluted at 1:100 in PBS supplemented with 0.1% Tween-20 (PBS-T). Membranes were then washed with PBS-T and further incubated for 1 h at room temperature with a goat anti-rabbit IgG HRP-conjugated secondary antibody (Sigma-Aldrich Chemie GmbH, Buchs SG, Switzerland) diluted at 1:1,000 in PBS-T. Luminescence was then developed using a Western Bot Quantum kit (Witec, Luzern, Switzerland) following the manufacturer’s instructions, and visualization and quantification were performed in the G:Box device using the GeneSys software (Syngene, Cambridge, UK). After signal detection, the membrane was immediately stripped with Restore Western Blot Stripping Buffer (Thermo Scientific, Wilmington, DE, USA) following the manufacturer’s instructions. In the step to detect the total amount of mTOR, the membrane was subjected to a second round of hybridization, in which the primary antibody was a rabbit polyclonal anti-mTOR (Cell Signal Technology, Danvers, MA, USA) diluted at 1:100 in PBS-T. Results were expressed as ratios of the signal obtained with anti-phospho mTOR and total mTOR antibodies, respectively, and normalized to an internal gel reference, a reference sample used in all gels, was used to reduce variability between gels.

### Statistical analysis

Data were analyzed using Proc MIXED of SAS (Version 9.2, SAS Inst. Inc., Cary, NC). Data on average daily feed intake (**ADFI**) and gain-to-feed (**G:F**) ratio determined from days 7 to 28 of age were analyzed as a two-way ANOVA, with dietary treatment and farrowing series as the fixed effect and rescue deck as the experimental unit. The remaining data were analyzed as a two-way ANOVA, with dietary treatments (CON and CarArg), sex (females and castrates), the dietary treatment × sex interaction and farrowing series (eight levels) as the main factors. The sow was used as a random factor and the individual pig was the experimental unit. As the dietary treatment × sex interactions were rarely significant (*P* < 0.05), they were excluded from the final statistical model for most of the analyzed traits. The least squares means of dietary treatment and sex were calculated and presented in the tables. The PDIFF option with the Tukey adjustment was used to determine differences among treatment groups when a significant dietary treatment × sex interaction was present. With the Proc CORR, Pearson correlation coefficients were calculated between BtW, CRL, BMI, PI, plasma concentrations of GH, IGF-1, insulin, and average daily gain (**ADG**) of the entire postweaning period. Differences were considered statistically significant at *P* < 0.05, and tendencies were assumed at 0.05 ≤ *P* ≤ 0.10.

## Results

### Body morphology, growth performance, and eating behavior

The CRL, BMI, and PI of the 28-d-old piglets did not differ between dietary treatments but tended to differ (*P* ≤ 0.09) between sex (Table 1). Females displayed greater (*P* = 0.03) CRL, smaller (*P* = 0.04) PI and tended to have a lower (*P* = 0.09) BMI than castrates. Except for females tending (*P* = 0.06) to grow slower than castrates from birth to 24 h after birth, no treatment or sex effects were observed in terms of ADG, ADFI, and G:F ratio in the period from birth to weaning. Intake of creep feed from days 21 to 28 was negligible and subject to large variation within and between dietary treatments (data not shown).

**Table 1.** Effect of feeding an unsupplemented milk replacer (CON) or a milk replacer supplemented with L-carnitine and L-arginine from 7 to 28 d of age on body morphology, growth performance and feed intake of selected L-BtW piglets born from hyperprolific sows^1^

Item^2^	Dietary treatment		Sex		SEM	*P*-value^4^	
	CON	CarArg	Castrate	Female		Trt	Sex
Body morphology							
CRL, cm	25.0	24.4	24.2	25.1	0.38	0.18	0.03
Body mass index	17.2	17.4	17.9	16.7	0.61	0.72	0.09
Ponderal index	70.0	72.1	74.7	67.4	3.32	0.59	0.04
BW, kg							
At birth	1.05	1.05	1.05	1.05	0.025	0.96	0.93
Day 7	1.75	1.72	1.75	1.71	0.057	0.65	0.62
Day 28	4.56	4.49	4.78	4.27	0.227	0.81	0.12
ADG, g/d							
Day 7 to d 28	135	132	145	122	9.6	0.81	0.10
ADFI^3^, g DM/d	298	287	—	—	19.2	0.69	—

^1^CarArg supplemented with 0.5 g L-carnitine and 16.7 g L-arginine per kg of diet. Results are presented as least squares means of the main factors of dietary treatment, sex, and pooled SEM.

^2^CRL = crown rump length, defined as the distance between the crown of the head and the base of the tail of the piglets determined on the day of birth; $\;Body\;\;\;mass\;\;\;index=Birth\;\;\;weight\;\;\;(kg)/Crown\;\;\;crump\;\;\;length\;\;\;(cm^{2})$; $Ponderal\;\;\;index=Birth\;\;\;weight\;\;\;\;\left(kg\right)/Crown\;\;\;crump\;\;\;length\;\;\;\;(cm^{3})$; ADFI = average daily feed intake.

^3^Rearing pen was used as the experimental unit, and thus sex was omitted from the statistical model, and the least square means for sex were not estimated.

^4^Probability values for the effects of dietary treatment (Trt) and sex, and differences were considered statistically significant at *P* < 0.05, and tendencies were assumed at 0.05 ≤ *P* ≤ 0.10.

At the start of the grower period, the CarArg piglets were heavier (*P* < 0.01) because they grew faster (*P* = 0.02) and ingested more feed (*P* = 0.01) in the starter period compared with the CON piglets (Table 2). However, in the grower, finisher, and overall growth periods, dietary treatments had no effects on the growth performance. Females were lighter on day 7 of age (*P* = 0.03), tended (*P* ≤ 0.08) to be lighter at slaughter, to eat less in the grower, finisher, overall growth periods, and to be more feed efficient in the finisher and overall growth periods than the castrates. In addition, females spent less (*P* < 0.01) time eating than castrates, whereas feed intake per visit and number of daily feeder visits were similar.

**Table 2.** Effect of feeding an unsupplemented milk replacer (CON) or a milk replacer supplemented with L-carnitine and L-arginine (CarArg) from 7 to 28 d of age on ADG, ADFI, feed efficiency (G:F), and feeding behavior^1^

Item	Dietary treatment		Sex		SEM	*P* value^6^	
	CON	CarArg	Castrate	Female		Trt	Sex
BW, kg							
At birth	1.07	1.08	1.07	1.08	0.029	0.74	0.92
Day 7	1.76	1.84	1.88	1.72	0.046	0.18	0.03
Day 28	5.03	5.08	5.41	4.71	0.331	0.91	0.18
Start of starter period	8.73	8.60	8.39	8.94	1.214	0.93	0.74
Start of grower period	21.2	23.2	22.1	22.2	0.46	<0.01	0.84
Start of finisher period^2^	63.0	63.6	63.4	63.2	0.75	0.28	0.63
Slaughter (day 170 of age)	95.3	97.6	101.3	91.6	3.59	0.57	0.06
Starter period, weaning to 25 kg BW							
ADG^3^, kg/d	0.4	0.5	0.5	0.4	0.03	0.02	0.06
ADFI^4^, kg/d	0.65	0.76	0.72	0.70	0.224	0.01	0.26
G:F^5^, kg/kg	0.6	0.6	0.6	0.6	0.03	0.60	0.10
Grower period, 25 to 60 kg BW							
ADG, kg/d	0.8	0.8	0.8	0.8	0.03	0.85	0.05
ADFI, kg/d	1.79	1.80	1.89	1.70	0.05	0.90	0.01
G:F, kg/kg	0.4	0.4	0.4	0.5	0.01	0.55	0.48
Finisher period, 60 kg BW to slaughter							
ADG, kg/d	0.8	0.8	0.9	0.7	0.06	0.83	0.08
ADFI, kg/d	2.73	2.70	3.10	2.34	0.152	0.86	0.01
G:F, kg/kg	0.3	0.3	0.3	0.3	0.01	0.94	0.08
Overall growth period, weaning to slaughter							
ADG, kg/d	0.7	0.7	0.8	0.7	0.03	0.63	0.11
ADFI, kg/d	1.85	1.85	2.00	1.70	0.109	0.99	0.07
G:F, kg/kg	0.4	0.4	0.4	0.4	0.01	0.41	0.05
Feeding behavior							
Feed intake, g/visit	207	198	214	192	21.6	0.75	0.50
Feed intake, g/min	29	31	30	29	2.5	0.42	0.75
Time spent eating, min/d	71.0	66.4	74.7	62.7	2.27	0.14	< 0.01
Number of daily visits	15.9	16.4	16.2	16.2	1.0	0.72	0.98

^1^CarArg supplemented with 0.5 g L-carnitine and 16.7 g L-arginine per kg of diet. Results are presented as least squares means of the main factors of dietary treatment, sex, and pooled SEM.

^2^Trt × Sex interaction; *P* = 0.05.

^3^Average daily gain.

^4^Average daily feed intake.

^5^Gain-to-feed.

^6^Probability values for the effects of dietary treatment (Trt) and sex, and differences were considered statistically significant at *P* < 0.05, and tendencies were assumed at 0.05 ≤ *P* ≤ 0.10.

### Organ weights and ratios and carcass composition

At 28 d of age, CarArg piglets tended (*P* ≤ 0.10) to have a greater brain-to-STM weight ratio and ash content of the carcass than their CON counterparts (Table 3). Females had lighter (*P* = 0.08) carcasses, hearts, livers, and lungs and the brain-to-liver and brain-to-STM weight ratios were greater (*P* ≤ 0.07) compared to those of the castrates. The fat and energy contents of the female carcasses were lower (*P* ≤ 0.02), whereas the carcass protein and ash contents were greater (*P* ≤ 0.02) than those of the castrates.

**Table 3.** Effect of feeding an unsupplemented milk replacer (CON) or a milk replacer supplemented with L-carnitine and L-arginine (CarArg) from 7 to 28 d of age on organ weights and ratios as well as carcass gross chemical composition at 28 d of age^1^

Item	Dietary treatment		Sex		SEM	*P*-value^2^	
	CON	CarArg	Castrate	Female		Trt	Sex
Carcass weight, kg	2.70	2.57	2.92	2.35	0.185	0.54	0.04
Organ weight, g							
Heart	25.1	24.2	27.0	22.3	1.72	0.65	0.08
Liver	113.2	98.4	118.4	93.1	8.23	0.14	0.05
Kidneys	27.3	27.7	28.7	26.2	1.84	0.89	0.37
Spleen	7.3	7.1	7.8	6.7	0.84	0.83	0.37
Lungs	57.0	54.1	60.6	50.4	3.56	0.49	0.06
Adrenal glands	0.8	0.7	0.8	0.7	0.08	0.36	0.59
Brain	44.1	43.4	43.4	44.1	1.10	0.52	0.66
Brain-to-liver weight	0.4	0.5	0.4	0.5	0.03	0.21	0.05
Brain-to-STM weight	4.4	4.9	4.3	5.0	0.29	0.06	0.07
Gross chemical composition of the carcass							
Gross energy, MJ/kg	24.6	24.2	24.9	23.8	0.32	0.20	0.02
Crude protein, g/kg	566	584	554	596	8.1	0.11	0.02
Ether extract, g/kg	281	258	298	241	14.0	0.11	<0.01
Total ash, g/kg	121	126	117	130	3.2	0.10	<0.01

^1^CarArg supplemented with 0.5 g L-carnitine and 16.7 g L-arginine per kg of diet. Results are presented as least squares means of the main factors of dietary treatment, sex, and pooled SEM.

^2^Probability values for the effects of dietary treatment (Trt) and sex, and differences were considered statistically significant at *P* < 0.05, and tendencies were assumed at 0.05 ≤ *P* ≤ 0.10.

At 170-d of age, the adrenal glands weight was greater (*P* = 0.04) in CarArg than CON pigs (Table 4). The hearts of CON castrates tended to be lighter whereas the hearts of the CarArg castrates tended heavier than those of the females in the respective treatment groups (CON castrates vs. CON females, 312 vs. 386 g; CarArg castrates vs. CarArg females, 404 vs. 356 g; dietary treatment × sex interaction [*P* < 0.05]).

**Table 4.** Effect of feeding an unsupplemented milk replacer (CON) or a milk replacer supplemented with L-carnitine and L-arginine (CarArg) from 7 to 28 d of age on organ weights and ratios at slaughter at 170 d of age^1^

Item	Dietary treatment		Sex		SEM	*P* value^2^	
	CON	CarArg	Castrate	Female		Trt	Sex
Organ weight, g							
Heart^3^	349	380	358	371	10.4	0.05	0.16
Liver	1,513	1,617	1,603	1,527	47.5	0.11	0.30
Kidneys	287	296	291	292	15.2	0.64	0.99
Spleen	126	133	133	126	9.8	0.58	0.66
Lungs	541	559	542	558	17.9	0.44	0.56
Adrenal glands	4.6	4.9	4.8	4.6	0.17	0.04	0.24
Brain	96.8	99.2	98.2	97.9	1.54	0.25	0.88
Brain-to-liver weight	0.1	0.1	0.1	0.1	0.002	0.29	0.34
Brain-to-STM weight	0.2	0.2	0.3	0.2	0.008	0.76	0.42
Small intestine							
Length, m	19.4	18.7	18.4	19.7	1.55	0.76	0.60
Weight, kg	1.8	1.9	1.9	1.8	0.09	0.21	0.33
Large intestine							
Length, m	6.1	6.4	6.4	6.1	0.293	0.46	0.48
Weight, kg	1.3	1.4	1.4	1.3	0.064	0.13	0.25

^1^CarArg supplemented with 0.5 g L-carnitine and 16.7 g L-arginine per kg of diet. Results are presented as least squares means of the main factors of dietary treatment, sex, and pooled SEM.

^2^Probability values for the effects of dietary treatment (Trt) and sex, and differences were considered statistically significant at *P* < 0.05, and tendencies were assumed at 0.05 ≤ *P* ≤ 0.10.

^3^Trt × Sex interaction; *P* = 0.032.

### Plasma hormone and serum metabolite concentrations

In the 28-d-old piglets, no differences in the concentration of serum metabolites were found between the treatment groups or between sexes (Table 5). At 170 d of age, the glucose and urea levels were greater (*P* = 0.01) and lower (*P* = 0.02), respectively, in the CON compared with the CarArg pigs and the NEFA and urea concentrations were greater (*P* = 0.05) and lower (*P* < 0.01) in females compared with the castrates.

**Table 5.** Effect of feeding an unsupplemented milk replacer (CON) or a milk replacer supplemented with L-carnitine and L-arginine (CarArg) from 7 to 28 d of age on serum metabolite concentrations at day 28 of age and at slaughter at 28 to 170 d of age^1^

Item^2^	Dietary treatment		Sex		SEM	*P* value^3^	
	CON	CarArg	Castrate	Female		Trt	Sex
28 d of age							
Glucose, mM	5.54	5.08	4.63	5.99	0.537	0.38	0.12
NEFA, mM	0.42	0.41	0.44	0.40	0.118	0.93	0.79
Urea, mM	4.70	4.11	4.09	4.72	0.578	0.39	0.46
170 d of age							
Glucose, mM	9.58	7.32	8.50	8.40	0.711	0.01	0.91
NEFA, mM	0.25	0.40	0.18	0.47	0.104	0.22	0.05
Urea, mM	5.59	6.47	6.93	5.13	0.296	0.02	<0.00
GH, ng/mL	1.8	2.3	2.1	2.0	0.46	0.35	0.80
IGF-1, ng/mL	17.5	25.0	18.9	23.6	8.74	0.51	0.71
Insulin, pg/mL	248	341	356	234	101.8	0.48	0.44

^1^CarArg supplemented with 0.5 g L-carnitine and 16.7 g L-arginine per kg of diet. Results are presented as least squares means of the main factors of dietary treatment, sex, and pooled SEM.

^2^NEFA = non-esterified fatty acids; GH = growth hormone; IGF-1 = insulin-like growth factor 1.

^3^Probability values for the effects of dietary treatment (Trt) and sex, and differences were considered statistically significant at *P* < 0.05, and tendencies were assumed at 0.05 ≤ *P* ≤ 0.10.

### Properties of the semitendinosus muscle

Supplementation of the milk replacer with L-carnitine and L-arginine had no effect on morphometric traits, myofibers number and size (Supplementary Table S1), or mTOR phosphorylation of the STM of the 28-d-old piglets (Supplementary Figure S1). Except for a shorter (*P* = 0.04) STM in females than castrates, sex had no effect on the aforementioned traits. The CS and LDH activities were greater (*P* ≤ 0.05) in the STMl but not in the STMd of CarArg compared with CON piglets (Table 6). In the STMd of the CarArg piglets, the HAD:CS ratio tended (*P* = 0.09) to be greater than in the CON piglets, whereas it was lower (*P* = 0.03) in the STMl. Furthermore, the LDH:CS ratio was lower (*P* = 0.05) in the STMd of the CarArg than in the CON piglets. Females, compared with castrates, displayed a greater HAD:CS ratio in the STMl (*P* = 0.01). At 170 d of age the STM of the CarArg pigs was longer (*P* = 0.02). Overall, of the STM of females tended to be shorter (*P* = 0.05; Table 6). The HAD:CS ratio in the STMd differed between dietary treatments, with CON pigs displaying a greater (*P* = 0.05) ratios. In the STMl, the LDH:HAD ratio tended to be greater (*P* < 0.08) for the CarArg pigs compared with the CON pigs.

**Table 6.** Effect of feeding an unsupplemented milk replacer (CON) or a milk replacer supplemented with L-carnitine and L-arginine (CarArg) from 7 to 28 d of age on the morphometric traits and the metabolic enzyme activities of the semitendinosus muscle at 28 and 170 d of age^1^

Item^2^	Dietary treatment		Sex		SEM	*P* value^3^	
	CON	CarArg	Castrate	Female		Trt	Sex
28 d of age							
STM weight, g	10.6	9.9	10.8	9.7	0.78	0.42	0.30
STM length, cm	65.7	65.2	68.2	62.7	1.65	0.77	0.04
STM circumference, cm	61.1	60.8	62.5	59.4	1.99	0.88	0.29
Dark STM							
CS (× 10^-2^), µM/min	0.58	0.45	0.47	0.57	0.082	0.18	0.41
HAD, µM/min	0.31	0.33	0.31	0.33	0.014	0.25	0.39
LDH, µM/min	1.64	1.44	1.45	1.62	0.108	0.13	0.30
HAD:CS	63.8	84.1	83.4	64.6	9.57	0.09	0.19
LDH:CS	338	368	406	299	46.2	0.55	0.10
LDH:HAD	5.5	4.5	4.9	5.0	0.41	0.05	0.96
Light STM							
CS (× 10^-2^), µM/min	0.37	0.57	0.52	0.42	0.080	0.05	0.43
HAD, µM/min	0.19	0.23	0.21	0.22	0.025	0.16	0.82
LDH, µM/min	1.53	2.09	1.65	1.96	0.201	0.04	0.28
HAD:CS	48.9	41.3	25.5	64.8	6.76	0.03	0.01
LDH:CS	477	461	413	525	73.4	0.85	0.29
LDH:HAD	9.7	8.7	8.7	9.7	1.45	0.49	0.61
170 d of age							
STM weight^4^, g	410	418	408	420	14.2	0.67	0.56
STM length, cm	18.4	19.1	19.1	18.5	0.21	0.02	0.05
STM circumference^5^, cm	22.2	22.4	22.2	22.5	0.44	0.70	0.65
Dark STM							
CS (× 10^-2^), µM/min	0.13	0.12	0.13	0.12	0.009	0.51	0.91
HAD, µM/min	0.16	0.13	0.15	0.15	0.016	0.12	0.99
LDH, µM/min	5.25	4.95	5.19	5.02	0.364	0.52	0.73
HAD:CS	131	105	118	1,170	10.7	0.05	0.98
LDH:CS	4,868	4,637	4,720	4,784	693.8	0.80	0.95
LDH:HAD	39.3	44.3	40.6	43.1	5.89	0.52	0.78
Light STM							
CS (× 10^-2^), µM/min	0.12	0.11	0.11	0.11	0.007	0.25	0.92
HAD, µM/min	0.11	0.09	0.10	0.10	0.009	0.10	0.93
LDH, µM/min	5.19	5.23	5.27	5.15	0.114	0.81	0.50
HAD:CS	89.8	83.1	87.1	85.8	7.01	0.44	0.89
LDH:CS	4,635	5,185	5,017	48,037	403.7	0.31	0.73
LDH:HAD	53.6	63.8	61.3	56.2	4.14	0.08	0.42

^1^CarArg supplemented with 0.5 g L-carnitine and 16.7 g L-arginine per kg of diet. Results are presented as least squares means of the main factors of dietary treatment, sex, and pooled SEM.

^2^Probability values for the effects of dietary treatment (Trt) and sex, and differences were considered statistically significant at *P* < 0.05, and tendencies were assumed at 0.05 ≤ *P* ≤ 0.10.

^3^HAD = β-hydroxyacyl-CoA dehydrogenase, CS = citrate synthase, LDH = lactate dehydrogenase.

^4^Trt × Sex interaction; *P* = 0.05.

^5^Trt × sex interaction; *P* = 0.03.

### Intestinal morphology and function of the 28-d-old piglets

No effect of dietary treatment or sex on intestinal morphology was found in the 28-d old piglets (Supplementary Table S3).

### Carcass characteristics and meat quality of pigs slaughtered on day 170 of age

Carcass characteristics (Supplementary Table S4) and meat quality (Supplementary Table S5) did not differ between CarArg and CON pigs. In agreement with the lower BW at slaughter, the hot and cold carcasses were lighter (*P* ≤ 0.05), and the percentage of chilling loss was greater in females than in castrates (*P* = 0.02; Supplementary Table S5). In addition, the carcasses of the females were leaner due to the greater (*P* ≤ 0.01) proportions of the loin, ham, and shoulder and the lower (*P* ≤ 0.03) proportions of subcutaneous, back, and omental fat in the carcasses. Several meat quality traits also differed between females and castrates (Supplementary Table S5). The LT quality traits were not affected by the dietary treatment. The LT pH at 45 min, the LT temperature at 3 h, and the LT b*-value tended to be lower (*P* ≤ 0.08) in females compared with castrates, whereas the LT temperature at 24 h tended (*P* = 0.10). In addition, drip, thaw, and cooking loss were greater (*P* < 0.05) in females compared with castrates, while intramuscular fat content was lower (*P* ≤ 0.06). By contrast, the shear force of the cooked meat of the females was greater (*P* = 0.02) compared with that of the castrates.

## Discussion

The number of L-BtW pigs is increasing as a result of the extensive selection of a greater number of total born pigs per litter in modern sow breeds (Martineau and Badouard, 2009). Although it is currently not common practice due to legislation and lack of experience, artificial rearing in rescue decks has been proposed as a proper measure to manage especially L-BtW piglets from as early as day 3 of age (De Vos et al., 2014). L-BtW piglets display shorter suckling duration than their average sized littermates (Hawe et al., 2020), increasing the likelihood of ingesting insufficient amounts of energy and nutrients. Thus, in the present study, L-BtW piglets were selected for artificial rearing with ad libitum access to milk replacer to improve their growth rate. We have previously shown that the supplementation of milk replacers with L-carnitine was beneficial for STM maturation, and L-arginine enhanced activation of the protein synthesis pathway in the STM of young, artificially reared pigs (Madsen et al., 2018a, 2018b). However, the results of both studies were not conclusive in showing that one supplement had an advantage over the other. Furthermore, we previously observed that the Arg level in sow milk was substantially greater (13.0 g/kg DM) (Madsen et al., 2018b) than effective levels previously reported (Kim and Wu, 2004). For this reason, the Arg level was substantially elevated compared with our previous studies (Madsen et al., 2018a, 2018b). In addition, and in comparison, with our previous studies, the aim was to investigate if potential additive effect of L-carnitine and L-arginine in the birth-to-weaning period would prolong into the entire postweaning period until slaughter. Therefore, it was hypothesized that combining the two supplements in one dietary treatment would elicit an additive effect on growth in the last 21 d of the nursing period, through promotion of muscle development of L-BtW piglets, and possibly facilitate postweaning growth while improving carcass composition and meat quality.

### Pre- and postnatal growth performance and eating behavior

The piglets in the CarArg group were heavier than the CON piglets at the beginning of the grower period due to the greater ADFI and consequently greater ADG in the starter period. However, this effect may not be due to the supplementation as neither of the two compounds, L-carnitine and L-arginine, are considered feed intake stimulants. L-arginine is a known precursor for nitric oxide (Wu and Morris, 1998), stimulating glucose transport in muscle and mitochondrial biogenesis (Jobgen et al., 2006), and L-carnitine is known to promote growth by facilitating transportation of fatty acids to muscle tissue for oxidation (Owen et al., 1996; Keller et al., 2012). More likely it was a temporary, but yet unexplainable, increase in ADFI and ADG as no carryover effect was observed, as this difference in growth performance did not persist into the subsequent phases. This lack of carryover effect was in contrast to a previous study where increased growth was observed in the post, but not pre-weaning period of piglets reared by Arg-supplemented lactating sows (Krogh et al., 2016). In support of this, concentrations of GH, IGF-1, and insulin, all essential drivers of growth (Ogilvy-Stuart et al., 1998), were also not sufficiently different between groups at slaughter.

Lack of difference in growth performance from weaning to slaughter is also supported by results from a previous study (Pardo et al., 2013) using animals from the same research herd and showing that conventionally reared pigs from sows having high (>1.7 kg) and low average litter BtW (<1.3 kg), and which weaned 12.0 and 8.5 pigs, respectively, did not differ greatly in terms of postweaning growth performance. Thus, although it is not possible to directly compare the growth performance results of the current and the previous study (Pardo et al., 2013), it indicates that artificial rearing does not to a great extent impair the postweaning growth performance of L-BtW piglets. In this regard, the markedly lower weaning weight in this compared with the previous study (Pardo et al., 2013) (5.03 to 5.08 kg BW vs. 7.37 to 9.70 kg BW), should also be taken into account. Despite having a similar BtW between treatments in the present experiment, diet did not impact BW at slaughter, partially due to its large variability (CON: 77 to 124 kg BW and CarArg: from 82 to 118 kg BW). One possible explanation might be that BtW alone is not a good predictor for postweaning growth performance, as recently discussed by Douglas et al. (2016). Douglas et al. (2016) suggested that morphological traits such as BMI and abdominal circumference were more accurate predictors. In this study, BMI was more strongly correlated with the ADG of the entire postweaning period (0.37; *P* = 0.02) as compared with BtW (0.27; *P* = 0.06). With this in mind, and the notably large variation in BW at slaughter, BtW may not be an accurate factor to use when selecting pigs for artificial rearing.

### Effect of L-carnitine or L-arginine supplementation on characteristics of muscle

Consistent with results from previous studies, no effects of supplementation were found on myofiber-related properties on day 28 of age (Madsen et al., 2018a, 2018b). By contrast, the CarArg supplementation affected the metabolic properties of the STM. The enzymes CS and LDH are involved in the oxidative and glycolytic muscle metabolism, respectively, whereas HAD plays a major role in the β-oxidation of fatty acids (Lefaucheur et al., 2003). Generally, the LDH:CS ratio clearly shows that independent of the dietary treatments the STMl has a more pronounced glycolytic metabolism than the STMd that was more than 100 units lower. This is in line with the lack of oxidative type I myofibers in the STMl. In the STMl, the combination of L-carnitine and L-arginine increased both the overall oxidative and glycolytic capacities. However, the increase had no effect on this portion with respect to the proportion between oxidative and glycolytic capacity as the LDH:CS-ratio was similar in CarArg and CON pigs. Lefaucheur et al. (2003) proposed that an increase in the glycolytic capacity of a muscle is a marker for a greater muscle maturation in young pigs. Such an increase was observed when artificially reared pigs were fed a milk replacer supplemented solely with L-arginine (Madsen et al., 2018a, 2018b). However, the reason why combining L-carnitine and L-arginine had no effect on muscle maturation remains unknown.

In the STMd, the HAD:CS ratio tended to be greater in the CarArg than the CON pigs. This indicates that the relative importance of lipid β-oxidation with respect to the global oxidative capacity increased, which could be the effect of carnitine increasing fatty acid oxidation (Eder, 2009). These findings concur also with the numerically lower fat and greater protein content of the carcasses of the CarArg compared with CON pigs slaughtered on day 28 of age. By contrast, in the STMl the HAD:CS ratio decreased which, could be due to the fact that this muscle relies predominantly on the glycolytic pathway.

Using the LDH:CS ratio as indicator of the muscle glycolytic capacity, one can conclude that the STMd and STMl are similar and that both portions relied more on the glycolytic than the oxidative pathway. This is in line with the fact that with increasing age the glycolytic metabolism is predominant (Lefaucheur, 2001). At slaughter day 170, the HAD:CS ratio of the STMd was lower in CarArg than CON pigs which is the opposite of what we observed in the young pigs slaughtered on day 28 of age. The lower ratio can be explained by the numerically lower HAD activity whereas CS activity was unaffected. It is worthwhile mentioning that HAD and CS activities were regardless of the dietary treatment on average 54% and 75% lower in the STMl of 170- than 28-d-old pigs.

### Effect of supplementation on carcass composition and meat quality

The pre-weaning dietary treatment had no impact on the carcass composition and the meat quality. As also shown in previous studies from the same research herd (Bérard et al., 2010a; Pardo et al., 2013), in the present study a number of differences between sexes were observed in carcass composition, where all traits except carcass yield and belly percentage differed between castrates and females. The greater appetite of castrates during the finisher period ultimately leads to a greater carcass weight but also greater fat and lower lean meat percentages compared with females. These findings are consistent with the greater overall feed efficiency mentioned previously in the discussion. In addition, pork from females was less tender and had greater cooking and thaw losses, which is similar to the findings presented by Pardo et al. (Pardo et al., 2013), where at least tenderness could be linked to the reduced IMF also found in females (Karlsson et al., 1999).

### Effect of sex

With respect to sex, females were relative to their BtW, longer and displayed therefore a lower PI and BMI compared with the castrates. Furthermore, a greater brain-to-organ weight ratio was observed in females compared with castrates, suggesting that females are neurologically more mature at birth. This is in contrast to previous results showing that L-BtW females are less competitive when suckling compared with males (Amdi et al., 2013). However, with respect to differences between sexes it cannot be excluded that the findings are exclusive for L-BtW piglet from hyperprolific sows, and not a general observation. Both before and after weaning females were leaner than castrates, suggesting lesser fat deposition, which was likely caused by the numerically lower feed and therefore energy intake. This finding is supported by a previous study from the same research herd, where a lower fat deposition was observed in females compared with castrated males at 100 kg slaughter weight (Ruiz-Ascacibar et al., 2017). While the growth rate was not affected in the overall growth period, both in the entire period and sub-periods of postweaning, females ate less than the castrates but, in turn, had a numerically greater feed efficiency in all except the starter period. This is partly also in line with findings from the study performed in the same research herd, where females compared with castrates had lower feed intake from 60 to 100 kg BW (Ruiz-Ascacibar et al., 2017). In addition, muscle of females compared with males contained less IMF, likely linked to NEFA metabolism (Tor et al., 2021), while the lower water holding capacity could be linked faster temperature drop 3h post mortem as a consequence of proteolysis (Schäfer et al., 2002). These effects of sex are in line with observations in previous studies (Gjerlaug-Enger et al., 2010; Szulc et al., 2018). Except for a dietary treatment and sex interaction with respect to BW at the start of the finisher period, no other interactions were observed indicating that the supplementary treatment was equally inefficient in promoting growth in both females and castrates.

## Conclusions

The combination of the dietary supplements L-carnitine or L-arginine to the milk replacer did not promote pre- or postweaning growth of artificially reared L-BtW piglets from large litters. In addition, carcass and meat quality were not positively affected by the supplements. On the contrary, sex had an effect on growth performance, carcass characteristic and meat quality traits in the favor of females over castrates. This is a known effect and appears to be independent of the dietary supplements in question as evidenced by the lack of interaction.

## Supplementary Material

skae122_suppl_Supplementary_Materials

## Abbreviations

L-BtW: , low-birth weight
BW: body weight
STM: semitendinosus muscle
STMd: semitendinosus dark portion
STMl: semitendinosus light portion
IUGR: intrauterine growth restriction
CRL: crown-to-rump length
BMI: body mass index
PI: Ponderal index
LT: Longissimus thoracis
DM: dry matter
NEFA: non-esterified fatty acids
ELISA: enzyme-linked immunosorbent assay
GH: growth hormone
IGF-1: insulin-like growth factor 1
TNF: total number of myofibers
CS: citrate synthase
HAD: β-hydroxyacyl-CoA dehydrogenase
LDH: lactate dehydrogenase
mTOR: Mammalian target of rapamycin
ADFI: average daily feed intake
G:F: gain-to-feed
ADG: average daily gain
