# Supplementary material for: Effect of sex and milk replacer with or without supplemental carnitine and arginine on growth characteristics, carcass, and meat quality of artificially reared low-birth weight pigs
Source: J Anim Sci. 2024 Jun 1;102:skae122. doi: 10.1093/jas/skae122 (PMC11143478; doi:10.1093/jas/skae122)
Supplement: skae122_suppl_Supplementary_Materials [file skae122_suppl_supplementary_materials.zip › Supplementary tables and figures.docx]

Supplementary tables and figures

**Supplementary Table S 1.** Analyzed nutrient composition (per kg DM) of milk replacer and post-weaning diets.

|  | Milk replacer^1^ | |  |  |  |
| --- | --- | --- | --- | --- | --- |
| Item | CON | CarArg | Starter diet^2^ | Grower diet^3^ | Finisher diet^4^ |
| Dry matter, g/kg | 966 | 965 | 896 | 890 | 889 |
| Total ash | 76.9 | 78.4 | 51.3 | 59.4 | 45.9 |
| Crude fat extract | 93.7 | 94.8 | 70.0 | 39.1 | 38.6 |
| Crude protein | 231 | 235 | 189 | 213 | 178 |
| Gross energy, MJ/kg DM | 18.6 | 18.6 | 19.1 | 18.5 | 18.6 |
| Amino acids, g |  |  |  |  |  |
| Alanine | 28.2 | 8.77 | 7.9 | 8.9 | 7.2 |
| Arginine | 7.1 | 16.7 | 9.8 | 13.6 | 8.9 |
| Aspartic acid | 17.0 | 17.2 | 16.6 | 20.8 | 14.9 |
| Cysteine | 4.8 | 4.8 | 3.3 | 3.6 | 3.4 |
| Glutamic acid | 42.0 | 42.3 | 31.9 | 42.2 | 33.4 |
| Glycine | 5.7 | 5.7 | 7.9 | 9.0 | 7.7 |
| Histidine | 4.4 | 4.4 | 4.2 | 5.8 | 4.0 |
| Isoleucine | 10.0 | 10.1 | 7.6 | 9.1 | 7.2 |
| Leucine | 17.8 | 18.0 | 15.4 | 16.2 | 14.3 |
| Lysine | 16.8 | 17.5 | 12.5 | 11.5 | 8.9 |
| Methionine | 5.1 | 5.0 | 3.2 | 3.3 | 3.1 |
| Phenylalanine | 8.6 | 8.6 | 9.6 | 11.1 | 9.8 |
| Proline | 14.7 | 14.8 | 12.3 | 14.3 | 14.5 |
| Serine | 9.8 | 9.9 | 8.6 | 10.1 | 8.2 |
| Threonine | 12.0 | 12.0 | 7.7 | 8.4 | 7.4 |
| Tryptophan | 3.47 | 3.5 | 2.6 | 2.9 | 2.6 |
| Tyrosine | 6.3 | 6.3 | 7.3 | 7.8 | 6.9 |
| Valine | 11.1 | 11.4 | 9.4 | 10.3 | 9.2 |

Commercial milk replacer Provimi Neopigg Rescuemilk 2.0 (Provimi BV, Rotterdam, The Netherlands) supplemented with either 20.5 g l -alanine (CON) or 0.5 g l-carnitine + 12.1 g l-arginine (CarArg) per kg.

2 Starter diet (weaning to 25 kg BW); main ingredients: barley, corn, oatmeal, wheat starch, and soybean meal). Supplied per kg of diet: 20 mg Cu; 100 mg Fe; 50 mg Mn; 275 mg Zn; 0.75 mg I; 0.75 mg Se; 20,000 IU vitamin A; 2,000 IU vitamin D3; 100 mg choline; 10 mg vitamin B1; 15 mg vitamin B2; 75 mg vitamin B_3_; 75 mg vitamin B_5_; 15 mg vitamin B_6_; 0.005 mg vitamin B_8_; 2.5 mg vitamin B_9_; 0.1 mg vitamin B_12_; 325 mg vitamin E; 5 mg vitamin K_3_.

3 Grower diet (25 to 60 kg BW); main ingredients: barley, soybean meal, and wheat starch). Supplied per kg of diet: 5.4 mg Cu; 27,2 mg Fe; 13.6 mg Mn; 74.5 mg Zn; 0.20 mg I; 0.20 mg Se; 10831 IU vitamin A; 4.1 mg of vitamin B_2_; 4.1 mg vitamin B_6_; 0.03 vitamin B_12_; 542 IU vitamin D_3_; 88.0 mg vitamin E; 1.40 mg vitamin K_3_; 270.8 mg choline; 0.07 mg of biotin; 20.4 mg of pantothenic acid; 0.69 mg of folic acid.

^4^ Finisher diets (60 kg BW to slaughter); main ingredients: barley, potato protein, and wheat). Supplied per kg of diet: 5.4 mg Cu; 27,2 mg Fe; 13.6 mg Mn; 74.5 mg Zn; 0.20 mg I; 0.20 mg Se; 10831 IU vitamin A; 4.1 mg of vitamin B_2_; 4.1 mg vitamin B_6_; 0.03 vitamin B_12_; 542 IU vitamin D_3_; 88.0 mg vitamin E; 1.40 mg vitamin K_3_; 270.8 mg choline; 0.07 mg of biotin; 20.4 mg of pantothenic acid; 0.69 mg of folic acid.

**Supplementary Table S 2.** Effect of feeding an unsupplemented milk replacer (CON) or a milk

replacer supplemented with l-carnitine and l-arginine (CarArg) from 7-28 days of age on the

myofibre characteristics of the semitendinosus muscle at 28 day of age^1^.

| Item | Dietary treatment | |  | Sex | |  | *P*-value^2^ | |
| --- | --- | --- | --- | --- | --- | --- | --- | --- |
|  | CON | CarArg |  | Castrate | Female | SEM | Trt | Sex |
| Total fiber number (× 10^3^) | 674 | 701 |  | 664 | 710 | 61.1 | 0.71 | 0.60 |
| Dark STM |  |  |  |  |  |  |  |  |
| Area, µm^2^ |  |  |  |  |  |  |  |  |
| Type I | 530 | 516 |  | 557 | 490 | 38.8 | 0.77 | 0.26 |
| Type II | 530 | 524 |  | 551 | 504 | 39.1 | 0.89 | 0.43 |
| Light STM^5^ |  |  |  |  |  |  |  |  |
| Area, µm^2^ |  |  |  |  |  |  |  |  |
| Type I | 367 | 328 |  | 368 | 326 | 27.8 | 0.25 | 0.31 |
| Type II | 425 | 394 |  | 437 | 382 | 33.2 | 0.44 | 0.26 |

CarArg supplemented with 0.5 g l-carnitine and 16.7 g l-arginine per kg of diet. Results are presented as least squares means of the main factors of dietary treatment, sex, and pooled SEM.

2 Probability values for the effects of dietary treatment (Trt) and sex, and differences were considered statistically significant at P < 0.05, and tendencies were assumed at 0.05 ≤ P ≤ 0.10.

**Supplementary Table S 3.** Effect of feeding an unsupplemented milk replacer (CON) or a milk

replacer supplemented with l-carnitine and l-arginine (CarArg) from 7-28 days of age on small and

large intestine morphology and functional traits of the small intestine on d 28 of age^1^.

| Item | Dietary treatment | | |  | Sex | |  | *P*-value^2^ | |
| --- | --- | --- | --- | --- | --- | --- | --- | --- | --- |
|  | CON | CarArg | |  | Castrate | Female | SEM | Trt | Sex |
| Small intestine | | |  |  |  |  |  |  |  |
| Length, m | 7.94 | 8.15 | |  | 8.18 | 7.91 | 0.352 | 0.61 | 0.60 |
| Weight, g | 168 | 182 | |  | 186 | 163 | 14.3 | 0.39 | 0.28 |
| Colon | | |  |  |  |  |  |  |  |
| Length, m | 1.45 | 1.50 | |  | 1.51 | 1.44 | 0.075 | 0.54 | 0.52 |
| Weight, g | 52 | 55 | |  | 55 | 52 | 0.1 | 0.62 | 0.60 |
| Intestinal morphology^3^ | | |  |  |  |  |  |  |  |
| Villi height^3^, μm | 363 | 347 | |  | 366 | 343 | 17.0 | 0.44 | 0.35 |
| Villi width^3^, μm | 146 | 141 | |  | 149 | 138 | 5.2 | 0.28 | 0.13 |
| Crypt depth^3^, μm | 139 | 133 | |  | 133 | 139 | 5.8 | 0.46 | 0.51 |

^1^ CarArg supplemented with 0.5 g l-carnitine and 16.7 g l-arginine per kg of diet. Results are presented as least squares means of the main factors of dietary treatment, sex, and pooled SEM.

^2^ Probability values for the effects of dietary treatment (Trt) and sex, and differences were considered statistically significant at P < 0.05, and tendencies were assumed at 0.05 ≤ P ≤ 0.10.

^3^ Based on transverse section average of the samples collected, respectively, from the middle of the duodenum, jejunum, and ileum.

**Supplementary Table S 4.** Effect of feeding an unsupplemented milk replacer (CON) or a milk

replacer supplemented with l-carnitine and l-arginine (CarArg) from 7-28 days on carcass

characteristics at slaughter at 170 d of age^[[1]](#footnote-1)^.

| Item^[[2]](#footnote-2)^ | Dietary treatment | |  | Sex | |  | | *P*-value^[[3]](#footnote-3)^ | |
| --- | --- | --- | --- | --- | --- | --- | --- | --- | --- |
|  | CON | CarArg |  | Castrate | Female | | SEM | Trt | Sex |
| Hot carcass weight, kg | 76.5 | 78.6 |  | 81.9 | 73.2 | | 2.98 | 0.55 | 0.05 |
| Chilling loss, % | 2.64 | 2.71 |  | 2.52 | 2.83 | | 0.078 | 0.50 | 0.02 |
| Carcass yield^2^, % | 80.4 | 80.5 |  | 80.8 | 80.1 | | 0.44 | 0.83 | 0.26 |
| Lean meat^2^, % |  |  |  |  |  | |  |  |  |
| Total | 54.8 | 55.2 |  | 52.1 | 57.9 | | 0.74 | 0.49 | <0.01 |
| Loin | 25.8 | 26.3 |  | 24.9 | 27.2 | | 0.38 | 0.32 | <0.01 |
| Ham | 17.4 | 17.0 |  | 16.1 | 18.3 | | 0.37 | 0.39 | <0.01 |
| Shoulder | 11.6 | 11.9 |  | 11.2 | 12.3 | | 0.19 | 0.15 | <0.01 |
| Belly, % | 16.8 | 17.3 |  | 17.2 | 16.9 | | 0.37 | 0.22 | 0.48 |
| Subcutaneous fat^2^, % | 15.1 | 15.0 |  | 17.7 | 12.4 | | 0.60 | 0.72 | <0.01 |
| Back fat^2^, % | 9.15 | 9.12 |  | 10.89 | 7.39 | | 0.486 | 0.95 | 0.03 |
| Omental fat^2^, % | 1.66 | 1.47 |  | 1.90 | 1.22 | | 0.144 | 0.26 | <0.01 |

1 CarArg supplemented with 0.5 g l-carnitine and 16.7 g l-arginine per kg of diet. Results are presented as least squares means of the main factors of dietary treatment, sex, and pooled SEM.

2 Carcass yield = hot carcass weight expressed as a percentage of the BW at slaughter; total = sum of denuded loin, ham and shoulder weight expressed as a percentage of the cold carcass weight; loin = denuded loin weight expressed as a percentage of the cold carcass weight; ham = denuded ham weight expressed as a percentage of the cold carcass weight; shoulder = denuded shoulder weight expressed as a percentage of the cold carcass weight; subcutaneous fat = sum of external fat weight from the loin, ham and shoulder expressed as a percentage of the cold carcass weight; back fat = external fat weight from the loin expressed as a percentage of the cold carcass weight; omental fat = omental fat weight expressed as a percentage of the cold carcass weight.

3 Probability values for the effects of dietary treatment (Trt) and sex, and differences were considered statistically significant at P < 0.05, and tendencies were assumed at 0.05 ≤ P ≤ 0.10.

**Supplementary Table S 5.** Effect of exchanging an unsupplemented milk replacer (CON) by a milk

replacer supplemented with l-carnitine and l-arginine (CarArg) from 7 to 28 d of age on meat quality

traits of the longissimus thoracis muscle at slaughter at 170 d of age^1^.

| Item | Dietary treatment | | |  | Sex | |  | *P*-value^2^ | |  |
| --- | --- | --- | --- | --- | --- | --- | --- | --- | --- | --- |
|  | CON | | CarArg |  | Castrate | Female | SEM | Trt | Sex |  |
| pH |  | |  |  |  |  |  |  |  |  |
| 45 min | 6.52 | | 6.62 |  | 6.63 | 6.51 | 0.052 | 0.11 | 0.08 |  |
| 3 h | 6.34 | | 6.47 |  | 6.48 | 6.32 | 0.079 | 0.18 | 0.16 |  |
| 24 h | 5.55 | | 5.56 |  | 5.58 | 5.53 | 0.029 | 0.96 | 0.29 |  |
| Temperature, °C | |  | |  |  |  |  |  |  |  |
| 45 min | 35.7 | | 36.2 |  | 35.6 | 36.3 | 0.46 | 0.15 | 0.15 |  |
| 3 h | 15.1 | | 15.6 |  | 16.1 | 14.7 | 0.52 | 0.41 | 0.05 |  |
| 24 h | 2.4 | | 2.6 |  | 2.3 | 2.7 | 0.18 | 0.39 | 0.10 |  |
| Color |  | |  |  |  |  |  |  |  |  |
| L* | 48.7 | | 48.4 |  | 48.9 | 48.2 | 0.60 | 0.58 | 0.26 |  |
| a* | 5.96 | | 5.33 |  | 5.72 | 5.56 | 0.380 | 0.22 | 0.78 |  |
| b* | 3.10 | | 3.16 |  | 3.26 | 3.02 | 0.127 | 0.32 | 0.06 |  |
| Water holding capacity, % | | | | | | | | | | |
| Drip loss | 1.74 | | 1.88 |  | 1.60 | 2.02 | 0.192 | 0.25 | 0.06 |  |
| Thaw loss | 9.56 | | 10.07 |  | 8.99 | 10.64 | 0.441 | 0.13 | 0.02 |  |
| Cooking loss | 21.65 | | 20.88 |  | 20.44 | 22.09 | 0.406 | 0.14 | 0.04 |  |
| Intramuscular fat, % | 3.82 | | 3.63 |  | 4.50 | 2.94 | 0.293 | 0.62 | <0.01 |  |
| Shear force, kg | 6.9 | | 6.4 |  | 5.9 | 7.4 | 4.14 | 0.24 | 0.02 |  |

^1^ CarArg supplemented with 0.5 g l-carnitine and 16.7 g l-arginine per kg of diet. Results are presented as least squares means of the main factors of dietary treatment, sex, and pooled SEM.

^2^ Probability values for the effects of dietary treatment (Trt) and sex, and differences were considered statistically significant at P < 0.05, and tendencies were assumed at 0.05 ≤ P ≤ 0.10.

**Supplementary Figure S 1.** Effect of supplementation with l-carnitine and l-arginine (CarArg; light grey bar) compared with the control (CON: dark grey bar) and the effect of sex (females: white bar and castrates: black bar) on the degree of phosphorylation of the Ser_2481_ residue on the mammalian target of the rapamycin (mTOR) pathway in the dark (STM dark) and light (STM light) portions of the semitendinosus muscle of the 28-day old piglets. Results are presented as the expression of phosphorylated mTOR relative to the total mTOR content, where each bar represents the least squares mean of each dietary tr

1. [↑](#footnote-ref-1)
2. [↑](#footnote-ref-2)
3. [↑](#footnote-ref-3)
