# Supplementary figures and images for: Effect of sex and milk replacer with or without supplemental carnitine and arginine on growth characteristics, carcass, and meat quality of artificially reared low-birth weight pigs
Source: J Anim Sci. 2024 Jun 1;102:skae122. doi: 10.1093/jas/skae122 (PMC11143478; doi:10.1093/jas/skae122)

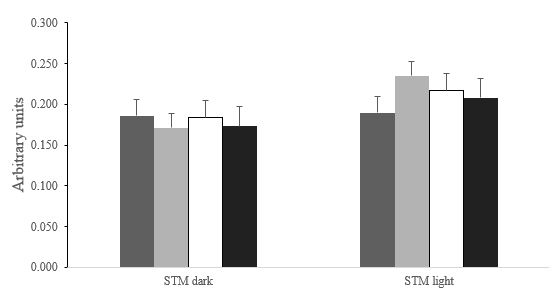

Supplement: skae122_suppl_Supplementary_Materials [file skae122_suppl_supplementary_materials.zip › Supplementary Figure S 1.JPG]
